# Identification of noble candidate gene associated with sensitivity to phytotoxicity of etofenprox in soybean

**DOI:** 10.1038/s41598-022-19323-0

**Published:** 2022-09-02

**Authors:** Ji-Min Kim, Jungmin Ha, Ilseob Shin, Ju Seok Lee, Jung-Ho Park, Jeong-Dong Lee, Sungteag Kang

**Affiliations:** 1 Department of Crop Science and Biotechnology, Dankook University, Cheonan, 31116 Korea; 2 Department of Plant Science, Gangneung-Wonju National University, Gangneung, 25457 Korea; 3 Bio-Evaluation Center, Korea Research Institute of Bioscience and Biotechnology, Cheongju, 28116 Korea; 4 School of Applied Biosciences, Kyungpook National University, Daegu, 41566 Republic of Korea

**Keywords:** Genetics, Molecular biology, Plant sciences

## Abstract

Phytotoxicity is caused by the interaction between plants and a chemical substance, which can cause critical damage to plants. Understanding the molecular mechanism underlying plant-chemical interactions is important for managing pests in crop fields and avoiding plant phytotoxicity by insecticides. The genomic region responsible for sensitivity to phytotoxicity of etofenprox (PE), controlled by a single dominant gene, was detected by constructing high density genetic map using recombination inbred lines (RILs) in soybean. The genomic region of ~ 80 kbp containing nine genes was identified on chromosome 16 using a high-throughput single nucleotide polymorphism (SNP) genotyping system using two different RIL populations. Through resequencing data of 31 genotypes, nonsynonymous SNPs were identified in *Glyma.16g181900*, *Glyma.16g182200*, and *Glyma.16g182300*. The genetic variation in *Glyma.16g182200*, encoding glycosylphosphatidylinositol-anchored protein (GPI-AP), caused a critical structure disruption on the active site of the protein. This structural variation of GPI-AP may change various properties of the ion channels which are the targets of pyrethroid insecticide including etofenprox. This is the first study that identifies the candidate gene and develops SNP markers associated with PE. This study would provide genomic information to understand the mechanism of phytotoxicity in soybean and functionally characterize the responsive gene.

## Introduction

Because of global warming, the major pests of soybean [*Glycine max* (L.) Merr.] have shifted from foliage-feeding coleopteran and lepidopteran pests to sap-sucking hemipteran pests during the last few decades^1^. Various stink bugs and soybean aphid species are the main hemipteran pests that damage soybean plants by sucking the juice from plant tissues^2^. Significant losses in soybean yield, quality, and germination have been caused by stink bug feeding^3^. The soybean aphid can cause up to 58% yield loss, and a $2.4 billion loss has been estimated annually in the US^4^.

Although synthetic pyrethroid insecticides have been widely used to manage insects, they might also harm plants. Phytotoxicity is defined as a harmful effect on various physiological processes. In various crop species including soybean, the several studies on interactions between plant physiology and pesticides have been reported^5–9^. The symptoms of phytotoxicity differ, including leaf speckling, leaf margin necrosis (browning) or chlorosis (yellowing), brown or yellow leaf spots, leaf cupping or twisting, plant stunting, and plant death^10^.

The insecticidal mode of action of pyrethroids, which derive etofenprox, relies on their ability to bind to voltage-gated sodium channels, disrupting insects’ nerve system^11^. Voltage-gated sodium channels are the most effective targets for the neurotoxic effects of pyrethroids, while voltage-gated chloride, and calcium channels work as secondary sites of action for a subset of pyrethroids^11,12^. Although the insecticide of etofenprox has been widely used in soybean fields to manage pests, few physiological and genetic studies of interactions between plants and pesticides have been reported. In a previous study, we firstly reported a novel trait, sensitivity to phytotoxicity of etofenprox (PE) in soybean, and it was illustrated that it was controlled by a single dominant gene through genetic analysis^13^. Only a limited number of genotypes, Danbaek and Kwangan, indicated leaf shrinkage due to the etofenprox application^13^. PE in Danbaek and Kwangan was regulated by the same gene inherited from a common ancestor, Tohoku 69^13^.

Because research tools have been rapidly developed, especially high-throughput genotyping systems (eg., Axiom SoyaSNP array), genomic research of soybean has been widely reported^14–18^. For example, single genes could be associated with flowering (*Glyma.10g221500*) and pod shattering (*Glyma.16g141600*), based on high throughput single nucleotide polymorphisms (SNPs) data in soybean^15,16^. Using genotypic data of Korean soybean collection comprising 1957 domesticated and 1079 wild accessions, domestication and evolutionary history were studied^17^. Through Axiom SoyaSNP array data of 2782 soybean collections, the core collection was developed for further genome-wide association study (GWAS)^18^.

The main goal of this study was to identify candidate genes responsible for sensitivity to PE using genomic approaches in soybean. To achieve this goal, a high saturated genetic map was constructed using a recombinant-inbred line (RIL) population, and a validation study was used with different RIL populations using TaqMan based SNP marker. Furthermore, whole-genome resequencing data from diverse soybean germplasm was used to develop SNPs in candidate genes associated with PE.

## Results

The sensitivity of PE was identified as a qualitative trait regulated by a single dominant gene^13^. In this study, two RIL populations were used to identify candidate genes and validate the results of genetic mapping. Among the 113 RIL populations derived from Daepung (insensitive) × Danbaek (sensitive), 79 lines showed sensitivity to PE and 34 lines showed insensitivity to PE. Among the 138 RIL population derived from 5002T (insensitive) × Kwangan (sensitive), 83 and 55 lines showed sensitivity and insensitivity to PE, respectively. Both populations exhibited discrete phenotypic distribution indicating PE is a qualitative trait.

**Figure 1:**
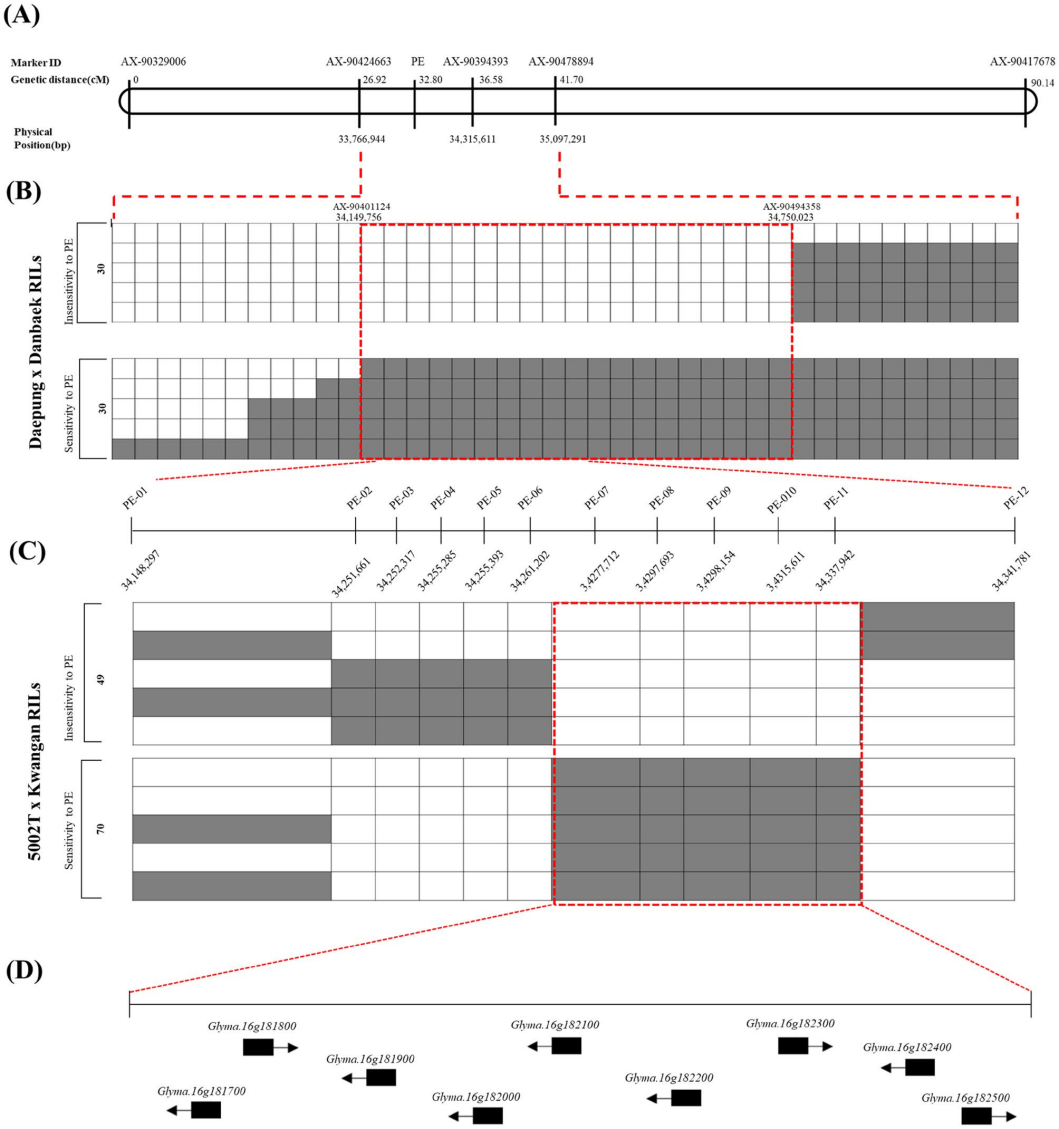
The genetic linkage map and its physical region of the locus conferring PE, which was identified by genetic mapping, on chromosome 16 from two RIL population. (**A**) The locus related to PE on the chromosome 16 from Daepung × Danbaek RILs population. (**B**) The genotypes distribution analysis for candidate region using RILs with extreme phenotypic data. The red box shows candidate region of PE. (**C**) Validation of candidate region using the twelve TaqMan base SNP markers in 5002T × Kwangan RIL population. (**D**) The nine candidate genes based on the reference genome of soybean Wm82.a2.v1^21^.

A highly saturated genetic map was constructed using Axiom 180K SoyaSNP array in the Daepung × Danbaek population (Fig. 1A, Fig. S1, Table S1). Out of 170,223 markers identified from the chip analysis, 9712 markers were used to construct 20 linkage groups, comprising 485 markers per linkage group, on average. Among the 1715 SNPs from the chip analysis, 358 SNP markers were used to construct chromosome 6 of the genetic map (Fig. S1, Table S1). The genomic region of 22.8 cM conferring 7.6 Mbp (Gm06:14,359,496..21,913,046) was associated with the color of pubescence and the *T* gene (Gm06:18,731,136..18,737,982) was located in this region. To construct chromosome 13 of the genetic map, 881 SNPs out of 1953, were used (Fig. S1, Table S2). The genomic region of 2.5 cM, corresponding to 29 Kbp (Gm13:17,365,410..17,336,628) was associated with the color of flower and *W1* gene (Gm13:17,312,425..17,317,057) was 20 Kbp apart from this region (Fig. S1, Table S2).

Out of 1075 SNP markers, 166 SNP markers were used to construct chromosome 16 (Fig. S1, Table S1). The genomic region of 9.6 cM was associated with PE, which was 548 kbp (Gm16:33,766,944..34,315,611). Genotype distribution analysis was conducted using 20 RILs showing extremely sensitive and insensitive phenotypes to refine the candidate region. Around two markers of AX-90424663 and AX-90394393, 29 polymorphic SNP markers were eliminated during the map construction because of redundancy. Through genotype analysis, including those unmapped SNP markers, the left and right borders of the candidate region were shifted from Gm16:33,766,944..34,315,611 to Gm16:34,149,756..34,750,023 (Fig. 1B). Thus, the region between AX-90401124 and AX-90494358 (~ 600 kbp, Gm16:34,149,756..34,750,023) exhibited clear discrimination of genotype distribution between the two groups, extremely sensitive and insensitive to PE (Fig. 1B).

To validate the results, 138 5002T × Kwangan RIL population was genotyped, where 83 and 55 lines showed sensitivity and insensitivity to PE, respectively, and the sensitivity of PE was scored. Around the candidate region identified using the Daepung × Danbaek RIL population, the genomic region between two markers, PE-07 and PE-11, showed clear discrimination of haplotypes, narrowing down the candidate region to ~ 80 kbp related to PE (Fig. 1C, Fig. S2). The results indicates that this region (~ 80 kbp, Gm16:34,261,202..34,341,781) appears to play a key role in soybean sensitivity to PE (Fig. 1C, Fig. S2).

Based on the Williams 82 genome assembly (https://soybase.org/gb2/gbrowse/glyma.Wm82.gnm4/ accessed on 18 January 2021), nine gene models were located with the candidate genomic region of ~ 80 kbp region related to PE^21^ (Fig. 1D). Out of nine annotated genes, 25 SNPs among Danbaek, Kwangan and Daepung were identified on the eight genes (Table 1). Among 25 SNPs, 15 SNPs resulted in nonsynonymous substitutions, located on the seven genes, including *Glyma.16g181700*, *Glyma.16g181900*, *Glyma.16g182100*, *Glyma.16g182200*, *Glyma.16g182300*, *Glyma.16g182400* and *Glyma.16g182500* (Table 1).

**Table 1:** Nine annotated gene models and SNPs in the exon of the genes in candidate gene regions between Danbaek, Kwangan and Daepung.

ID	Gene name	Protein function	Position(bp)	SNP^a^	Amino acid change^b^
SNP-01	*Glyma.16g181700*	C2H2-LIKE ZINC FINGER PROTEIN	34,258,523	A/G	–
SNP-02			34,261,202	T/C	Gln → Arg
-	*Glyma.16g181800*	–	–	–	–
SNP-03	*Glyma.16g181900*	Kinesin like protein	34,277,712	G/A	Gly → Asp
SNP-04	*Glyma.16g182000*	OXOGLUTARATE/IRON-DEPENDENT OXYGENASE	34,286,789	C/T	–
SNP-05	*Glyma.16g182100*	CGI-141-RELATED/LIPASE CONTAINING PROTEIN	34,290,385	A/T	–
SNP-06			34,291,832	C/A	Ala → Glu
SNP-07	*Glyma.16g182200*	GPI-anchored adhesin-like protein	34,297,693	A/C	Lys → Thr
SNP-08			34,299,069	G/T	Arg → Leu
SNP-09			34,299,443	C/T	–
SNP-10			34,300,135	T/C	Tyr → His
SNP-11			34,301,510	G/A	–
SNP-12			34,301,933	A/G	Iso → Met
SNP-13	*Glyma.16g182300*	Cleavage and polyadenylation specificity factor subunit 2 (CPSF2, CFT2)	34,310,819	C/T	–
SNP-14			34,315,218	C/T	Thr → Ile
SNP-15	*Glyma.16g182400*	PROTEIN NLP6-RELATED	34,323,857	C/T	Ser → Phe
SNP-16			34,324,632	T/C	–
SNP-17			34,325,885	C/T	Ala → Val
SNP-18			34,326,712	C/T	Thr → Met
SNP-19			34,326,832	T/A	Leu → Gln
SNP-20			34,326,888	C/A	–
SNP-21	*Glyma.16g182500*	Tripeptidyl-peptidase II	34,332,051	G/T	Arg → Leu
SNP-22			34,335,999	C/T	–
SNP-23			34,337,942	T/C	Leu → Ser
SNP-24			34,339,680	A/G	Asp → Arg
SNP-25			34,345,675	T/C	–

^a^Reference allele/alternative allele.

^b^Reference amino acid → alternative amino acid.

To confirm the genetic variations within the seven gene models, nucleotide sequences of the genes were compared among 31 soybean genotypes including Danbaek and Kwangan. Several germplasms of insensitivity to PE had the same alleles as Danbaek and Kwangan at SNP-01, 03, 05, 06, 07, 09, 10, 11, 12, 13, 14 and 15 (Table 2). However, all genotypes with insensitivity to PE had distinct alleles from Danbaek and Kwangan at SNP- 02, 04 and 08, located between 34,277,712 and 34,315,218 bp on soybean chromosome 16 (Table 2). These three SNPs cause amino acid substitutions in three genes, and it is proposed that these three genes, *Glyma.16g181900*, *Glyma.16g182200*, and *Glyma.16g182300*, are promising candidate genes associated with PE in soybean.

**Table 2:** SNPs within genes showed amino acid changes in diverse soybean germplasm.

Gene name	*Glyma.16g181700*	*Glyma.16g181900*	*Glyma.16g182100*	*Glyma.16g182200*				*Glyma.16g182300*
SNP name	SNP-01	SNP-02	SNP-03	SNP-04	SNP-05	SNP-06	SNP-07	SNP-08
Position (bp)	34,261,202	34,277,712	34,291,832	34,297,693	34,299,069	34,300,135	34,301,933	34,315,218
AA change	Gln- > Arg	Gly- > Asp	Ala- > Glu	Lys- > Thr	Arg- > Leu	Tyr- > His	Iso- > Met	Thr- > Ile
Danbaek^a^	C	A	A	C	T	C	G	T
Kwangan^a^	C	A	A	C	T	C	G	T
Williams82^b^	T	G	C	A	G	T	A	C
Daepung^b^	T	G	C	A	G	T	A	C
Bangsa^b^	T	G	A	A	T	C	G	C
Daewon^b^	T	G	C	A	H	T	A	C
Cheongja^b^	C	G	A	A	H	T	A	C
Cheongja3^b^	C	G	A	A	T	C	G	C
Daeheug^b^	C	G	A	A	T	C	G	C
Galchae^b^	C	G	A	A	T	C	G	C
Haman^b^	T	G	C	A	G	T	A	C
Hannam^b^	C	G	A	A	T	C	G	C
Heugchung^b^	C	G	A	A	G	T	A	C
Hwangeum^b^	T	G	C	A	G	T	A	C
Ilpumgeomjeong^b^	C	G	A	A	G	T	A	C
Josaengseori^b^	T	G	A	A	T	C	G	C
Pungwon^b^	C	G	A	A	G	T	A	C
Pureun^b^	T	G	C	A	G	T	A	C
Saedanbaek^b^	T	G	C	A	G	T	A	C
Seoritae^b^	C	G	A	A	T	C	G	C
Shinhwa^b^	T	G	A	A	T	C	G	C
Sochung2^b^	C	G	A	A	G	T	A	C
Soho^b^	T	G	A	A	T	C	G	C
Somyeong^b^	C	G	A	A	G	T	A	C
Taekwang^b^	T	G	C	A	G	T	A	C
Uram^b^	C	G	A	A	T	C	G	C
1000Al^b^	T	G	C	A	G	T	A	C
Iksan10^b^	T	G	A	A	T	C	G	C
Keunol^b^	C	G	A	A	T	C	G	C
Shinpaldal^b^	T	G	C	A	G	T	A	C
Yonpoong^b^	T	G	C	A	G	T	A	C

Gene name	*Glyma.16g182400*				*Glyma.16g182500*			
SNP name	SNP-9	SNP-10	SNP-11	SNP-12	SNP-13	SNP-14	SNP-15	
Position (bp)	34,323,857	34,325,885	34,326,712	34,326,832	34,332,051	34,337,942	34,339,680	
AA change	Ser- > Phe	Ala- > Val	Thr- > Met	Leu- > Gln	Arg- > Leu	Leu- > Ser	Asp- > Arg	
Danbaek^a^	T	T	T	A	T	C	G	
Kwangan^a^	T	T	T	A	T	C	G	
Williams82^b^	C	C	C	T	G	T	A	
Daepung^b^	C	C	C	T	G	T	A	
Bangsa^b^	C	T	C	T	G	T	A	
Daewon^b^	C	C	C	T	G	T	A	
Cheongja^b^	C	T	C	T	T	T	A	
Cheongja3^b^	C	T	C	T	T	C	G	
Daeheug^b^	C	T	C	T	T	C	G	
Galchae^b^	T	T	T	A	T	C	G	
Haman^b^	C	C	C	T	G	T	A	
Hannam^b^	T	T	T	A	T	C	G	
Heugchung^b^	T	T	T	A	T	C	G	
Hwangeum^b^	C	C	C	T	G	T	A	
Ilpumgeomjeong^b^	C	T	C	T	T	T	A	
Josaengseori^b^	T	T	T	A	G	T	A	
Pungwon^b^	C	T	C	T	G	T	A	
Pureun^b^	C	C	C	T	G	T	A	
Saedanbaek^b^	C	T	C	T	T	T	A	
Seoritae^b^	C	T	C	T	T	T	A	
Shinhwa^b^	C	T	C	T	G	T	A	
Sochung2^b^	C	T	C	T	T	T	A	
Soho^b^	C	T	C	T	G	T	A	
Somyeong^b^	C	T	C	T	G	T	A	
Taekwang^b^	C	T	C	T	T	T	A	
Uram^b^	C	T	C	T	T	C	G	
1000Al^b^	C	C	C	T	G	T	A	
Iksan10^b^	C	T	C	T	G	T	A	
Keunol^b^	C	T	C	T	T	C	G	
Shinpaldal^b^	C	C	C	T	G	T	A	
Yonpoong^b^	C	C	C	T	G	T	A	

^a^Sensitivity to PE.

^b^ Insensitivity to PE.

## Discussion

Through the 180K Axiom SoyaSNP assay, the high-density genetic map was constructed for the Daepung × Danbaek RIL population. In the population, the colors of flower and pubescence, which had been reported to be controlled by single genes previously^19,20^, were divided into purple (70) and white (43), gray (76), and brown (37), respectively. The responsive genes, T and W1, for the colors of pubescence and flower could be identified using the constructed genetic map.

The candidate region (~ 80 kbp) conferring PE was narrowed down from (kbp) to ~ 80 kbp using the second RIL population (5002T × Kwangan) through TaqMan based SNP assay. The genomic region contains nine annotated gene models on chromosome 16 based on the Williams 82 genome assembly (https://soybase.org/gb2/gbrowse/glyma.Wm82.gnm4/ accessed on 18 January 2021). Using high-depth resequencing data on 31 soybean germplasm, three candidate genes, *Glyma.16g181900*, *Glyma.16g182200* and *Glyma.16g182300* had amino acid substitutions between genotypes with insensitivity (29 genotypes) and sensitivity (Danbaek and Kwangan) to PE. *Glyma.16g182200* is the most promising candidate gene associated with sensitivity to PE, Therefore, accurately evaluated phenotypes, high-density genetic map, and genomic information from diverse germplasm would be essential for successful genetic mapping to identify candidate gene(s) of a target trait.

Arabidopsis homologous gene, *AT5G02370.1*, a homologous *Glyma.16g181900*, encodes adenosine triphoshpate binding microtubule motor family protein (https://www.arabidopsis.org/). In wild-type, trichomes have a stalk and three or four branches. In contrast, in *zwichel* (*zwi*) mutants, the trichomes have a shortened stalk and only two branches resulting in leaf senescence, similar to the morphological symptoms induced by PE^22^. *Glyma.16g182300* encodes cleavage and polyadenylation specificity factor 100 (CPSF 100), a homologue of *AT5G23880.1* in Arabidopsis. In Arabidopsis, CPSF100 mutant showed early flowering phenotypes induced by modified RNA processing of flowering control locus A enhancer of flowering^24,25^. Besides flowering time, the CPSF protein family is involved in various functions in biological processes such as environmental response and amino acid metabolism^23,26,27^. A variant of this gene might also affect the responses of plants to environmental stresses at the level of transcription, causing sensitivity to PE.

*AT5G23890.1*, Arabidopsis orthologue of *Glyma.16g182200*, encodes glycosylphosphatidylinositol (GPI)—anchored protein (GPI-AP), allowing various proteins to associate with membrane lipid bilayers and anchor on the external surface of the plasma membrane^28^. The GPI oligosaccharide structure is ubiquitous among eukaryotes with a common minimal backbone comprising three mannoses, one non-N-acetylated glucosamine, and an inositol phospholipid, which covalently links the carboxyl terminus (C terminus) of GPI-Aps to the lipid bilayer^29^. GPI-Aps include diverse families, such as cell wall structure proteins, proteases, enzymes, and lipid transfer proteins. They are involved in several functional processes, including cell wall composition, cell wall component synthesis, polar cell expansion, stress responses, hormone signaling responses, and pathogen responses in Arabidopsis^28^.

Voltage-gated sodium channels are important for initiating and propagating the action potential of neurons. Because of their essential roles in electrical signaling, sodium channels are primary targets of synthetic insecticides, including etofenprox. These sodium channel neurotoxins bind to the receptor on the sodium channel, changing different channel properties, including ion selectivity, ion conductance and/or channel opening and closing^12^. GPI-Aps have been reported to play roles in ion channels^30–32^.

TEX101, a germ cell-specific GPI-AP, belongs to the lymphocyte antigen 6 (Ly6)/urokinase-type plasminogen activator receptor (uPAR)-(LU) protein superfamily^33^. A recent crystal structure analysis of TEX101 provides evidence that this molecule has two LU domains, which function as a regulator of ion channels^34^. This structural feature holds great promise for elucidating the actual interactions between this molecule and a group of molecules associated with the ion channels^35^. In zebrafish, voltage-gated sodium channels are not located at the cell surface in the mutant of a subunit of GPI transamidase, which is crucial for membrane anchoring of GPI-anchored proteins. The biogenesis of GPI-anchored proteins is necessary for cell surface expression of sodium channels of neurons in zebrafish^35,36^.

The protein structure of the candidate gene was predicted using the AlphaFold Protein Structure Database (https://alphafold.ebi.ac.uk/) (Fig. S3)^37^. Comparing the predicted structures of *Glyma.16g182200*, the energy minimized prediction model showed the difference in length of residue between the Daepung and Danbaek types (Fig. S3B,C). Taken together, the PE symptoms in this study might have originated from the structural disruption at the active site of GPI-AP that is anchored on ion channels, which are the targets of pyrethroid insecticide, causing leaf shrinkage or senescence by cell wall disruption.

The SNP markers and high-resolution genetic map identified in the this study will facilitate MAS of PE in soybean. PE-sensitive genotype specific SNP markers were identified. Among the three candidate genes, *Glyma.16g182200*, encodes GPI-AP, is the most promising causal single gene regulating PE in soybean. Further functional characterization would be required through RNA expression analysis or cloning to transformation of the candidate gene. Identification of the responsive gene will improve our understanding of the basic mechanism of sensitivity to PE in soybean.

## Materials and methods

### Plant materials and the evaluation of three qualitative traits

Two recombinant inbred line (RIL) populations were used in the present study. The first population of RILs was developed from a cross between Daepung^38^ and Danbaek^39^. Daepung showed insensitivity to etofenprox and Danbaek showed sensitivity to etofenprox^13^. After artificial crossing in 2012, F_1_ progeny was advanced using single seed descent in the Dankook University greenhouse resulting in 119 F_5:8_ lines used for this study in 2019.

The second 138 RIL population was derived from a cross between 5002T^40^ showing insensitivity to etofenprox and Kwangan^41^ showing sensitivity to etofenprox, in the experimental field of Kyungpook National University. 5002T was developed for high yield with maturity group V in the southern USA in 2002^40^ and Kwangan was developed for high-protein^13^. This population was used to confirm genetic mapping and inheritance of sensitivity to PE with a different genetic background. Furthermore, from these two RIL populations, SNP markers associated with sensitivity to etofenprox were developed.

The RILs were sowed using a mixture of horticultural soil and the nursery bed soil at the ratio of 3: 1 in 50 deep cell seed trays, 55 × 27 × 12 cm (Wide × Length × Height). Etofenprox 20% EC 1000 × dilution was foliar sprayed four times in two weeks at V_1_ stage^13^. Distilled water was used as a negative control. A 10 mL was applied to each plant in each application and the phenotype (sensitivity/insensitivity) was evaluated by presence/absence of leaf shrinkage a week after the final treatment. The treatments were conducted with three biological replications^13^.

Flower color and pubescence color were also scored in two RIL populations for detecting *W1* and *T* genes, respectively, previously reported^19,20^. Three qualitative phenotypic data (sensitivity of PE, flower color, and pubescence color) were recorded by two types, reference allele or alternative allele.

### DNA extraction, SNP genotyping, and genetic map construction

Genomic DNA from each line and the parents of two RIL populations was extracted from young trifoliate leaves using the cetyltrimethylammonium bromide method^42^ with the following modifications : an incubation time of 90 min, re-suspension of the DNA pellet in 500 μL 1 × TE, and no RNase A treatment. First, all DNA was quantified using an ND-1000 Spectrophotometer and diluted to 100 ng µL^−1^ for further study.

For the SNP genotyping, Axiom 180K SoyaSNP assay (Affymetrix, CA, USA) was used^14^ in the Deapung × Danbaek RIL population. Genomic DNA from the lines and parents was hybridized into the Affymetrix GeneTitan array system and then scanned using GeneTitan Scanner (Affymetrix, CA, USA) following the manufacturer’s protocol. SNP genotype analysis was conducted based on Axiom Genotyping Solution Data Analysis User Guide (http://www.affymetrix.com)^43^.

The genetic map was constructed using the QTL ICIMapping software ver. 4.1^44^ according to the following parameters: binning by segregation distort 0.01, grouping by a 3.0 logarithm of odds threshold, nnTwoOpt (nearest neighbor with a two-opt heuristic algorithm) for ordering algorithm^45^, and rippling by the sum of adjacent recombination. The kosambi mapping function was used for the genetic map construction^46^. The genotype information of the unmapped SNP marker is used to narrow down the mapping region. Redundant markers were removed for map construction.

### TaqMan SNP genotyping assay and Sequence comparison with soybean germplasm

To validate the result of genetic mapping from the Deapung × Danbaek RIL population, the probes for the TaqMan assay were designed based on the twelve SNPs originating from Axiom 180K SoyaSNP assay (SFC-dye, South Korea) (Table S2). The extracted DNA was mixed with SFC Master Mix buffer, probes, and primers for the TaqMan SNP genotyping assay. The polymerase chain reactions (PCRs) were run on ABI Step one plus real-time PCR instrument (Applied Biosystems, CA, USA). The PCR conditions were initially 2 min at 50 ℃, 10 min at 95 ℃ for denaturation reaction, and 40 cycles (95 ℃ for 15 s and 60 ℃ for 1 min). At the end of each cycle, the fluorescence intensities of VIC and FAM were measured at the end of each cycle. The fluorescence intensity results were analyzed using the StepOne software V.2.3 (Applied Biosystems, CA, USA)^47^.

Sequence variations showing amino acid changes within the candidate genes were detected by mapping resequencing data of Danbaek, Kwangan and Daepung against soybean reference genomic sequence^21^. To confirm the sequences variation, diverse soybean germplasm, totally 31 soybean germplasm, including 25 parents for the Korean soybean nested association mapping populations, were used (Table 3)^48^. Out of 31 germplasm, two Korean soybean cultivars, Danbaek and Kwangan, present sensitivity to PE and the other 29 germplasms were insensitive to PE.

**Table 3:** Information of 31 soybean germplasms used for sequence comparison.

Name of varieties	Source	Breeding method	Name of varieties	Source	Breeding method
Daepung	Variety	Artificial crossing	Hwangkeum	Variety	Artificial crossing
Bangsa	Variety	Gamma-ray	Cheongja	Variety	Artificial crossing
Pungwon	Variety	Artificial crossing	Cheongja3	Variety	Artificial crossing
Hannam	Variety	Artificial crossing	Socheong2	Variety	Artificial crossing
Somyeong	Variety	Artificial crossing	Ilpumgeomjeong	Variety	Artificial crossing
Galchae	Variety	Artificial crossing	Daeheug	Variety	Artificial crossing
Soho	Variety	Artificial crossing	Josaengseori	Variety	Gamma-ray
Shinhwa	Variety	Artificial crossing	Yonpoong	Variety	Artificial crossing
Pureun	Variety	Artificial crossing	1000Al	Breeding line	Artificial crossing
Taekwang	Variety	Artificial crossing	Heugcheong	Variety	Artificial crossing
Uram	Variety	Artificial crossing	Seoritae	Landrace	–
Danbaek	Variety	Artificial crossing	Keunol	Variety	Artificial crossing
Haman	Landrace	–	Shinpaldal	Variety	Artificial crossing
Williams 82	Variety	Artificial crossing	Iksan10	Breeding line	Artificial crossing
Saedanbaek	Variety	Artificial crossing	Kwangan	Variety	Artificial crossing
Daewon	Variety	Artificial crossing			

### Protein structure prediction

The protein structure of the candidate gene was obtained from the AlphaFold Protein Structure Database (https://alphafold.ebi.ac.uk/)^37^. The previously identified nonsynonymous substitution was generated using the mutation function in Swiss PDB viewer 4.1.0 and energy minimization was conducted to obtain predicted protein structure in Danbaek. Both the predicted protein structures of Daepung and Danbaek were visualized and compared using USCF Chimera X software^49^.

### Ethical approval

The soybean cultivars ‘Danbaek’, ‘Kwangan’ and ‘Daepung’ were provided by National Agrobiodiversity Center in Jeonju, Korea. The soybean cultivar ‘5002T’ was provided by USDA Germplasm Resources Information Network (GRIN) database (https://www.ars-grin.gov). All the experiments carried out on plants in this study followed relevant institutional, national, and international guidelines and legislation.

## Supplementary Information

Supplementary Table S1.

Supplementary Information.

## Data Availability

The datasets and plant materials generated and analyzed during the current study are available from the corresponding author upon request. Raw reads in fastq format for all re-sequenced accessions to NCBI SRA with SRA accession number PRJNA555366^48^. Datasets of SNPs are available from figshare repository (https://figshare.com/projects/Soybean_haplotype_map_project/76110)^48^.
