# Supplementary material for: Identification of noble candidate gene associated with sensitivity to phytotoxicity of etofenprox in soybean
Source: Sci Rep. 2022 Sep 2;12:14944. doi: 10.1038/s41598-022-19323-0 (PMC9440009; doi:10.1038/s41598-022-19323-0)
Supplement: Supplementary file 2 — Supplementary Information. [file 41598_2022_19323_MOESM2_ESM.docx]

Table S2. Information of TaqMan based SNP markers used in validation of genetic mapping result

| Marker | Position | SNP information^a^ | Marker | Position | SNP information^a^ |
| --- | --- | --- | --- | --- | --- |
| PE-01 | 34,148,297 | F: GGGAAATTGCTCTCTTGGAAAA | PE-07 | 34,277,712 | F: GACTAAGTGAGTCAAACCTGAATTGC |
|  |  | R: GAAATCACACAGATTCTGCAAAGC |  |  | R: ACAGCACTGTTATTGCACATGGT |
|  |  | FAM : AGGAGCAAAGGACT |  |  | FAM : TCTTTCCACTATCCC |
|  |  | VIC : AGGAGCAAAAGACT |  |  | VIC : TCTTTCCACTACCCC |
| PE-02 | 34,251,661 | F: AATGCACCCTATCTTGTTTTTGC | PE-08 | 34,297,693 | F: CAATCACCAGCAACTCGCTTT |
|  |  | R: GGTCTTTCCTTCTCCTATTCTTCTTTT |  |  | R: AGTTGCAGCAAAATGCTCTTGA |
|  |  | FAM : CTTACCTTGTCTAAACAT |  |  | FAM : CACCTTCTGTGATACCAA |
|  |  | VIC : ACCTTGTCTAAAAATATGA |  |  | VIC : CACCTTCTTTGATACC |
| PE-03 | 34,252,317 | F: CCAGCATCAGTTGGTTATAGAATATCTT | PE-09 | 34,298,154 | F: TGACACATATATTGTCCAGTCCTAAAA |
|  |  | R: AAAAATGCACTAAACCAGTCAGGAA |  |  | R: CCGAGAATCAACATAACCATTTATT |
|  |  | FAM : AAATTCCATGGACCCC |  |  | FAM : AGCTGTTAGCCTATTAC |
|  |  | VIC : AATTCCGTGGACCCC |  |  | VIC : TAGCTGTTAGCGTATTAC |
| PE-04 | 34,255,285 | F: GAGCCGAGAACCTTTGAGATGA | PE-10 | 34,315,611 | F: TATATTGCATCCATTGAAACTTATAAGTTCT |
|  |  | R: ACCAGGAAGGGATCAAAGCA |  |  | R: TTCTTTTTTCAAAAAATCTACCTTAGTGTTTAT |
|  |  | FAM : CCACTCCCTCCATGAA |  |  | FAM : CTAATTCAGAATACAAACCT |
|  |  | VIC : ATGCCACTCCTTCCA |  |  | VIC : TCAGAATGCAAACCT |
| PE-05 | 34,255,393 | F: CTCCAACTTTATGCCCTCATAGGT | PE-11 | 34,337,942 | F: TGATTGCTAATGACAGGTCCTTCA |
|  |  | R: CCTGGTCACCCTCAAAAACAC |  |  | R: TGATCTCACCTCCGTAGATTGTTG |
|  |  | FAM : TGGCCTCTTCACTC |  |  | FAM : CATCTACCTCAGGGAA |
|  |  | VIC : ACCATTGGCCTTTT |  |  | VIC : ATCTACCTTAGGGAAGC |
| PE-06 | 34,261,202 | F: GCTGTCCCACATAAACAAGTATTTTC | PE-12 | 34,341,781 | F: GCTACAAACACATAAATACAGAAGAATTG |
|  |  | R: GCTTTCCTATATCTTCCCAGGAGTT |  |  | R: GCTATTGCCCTGTAGTCTTATTCTTTT |
|  |  | FAM : CATCATCAAGGATGCT |  |  | FAM : TCAATGTGAATATGGAATAA |
|  |  | VIC : CATCATCAAGGATACTG |  |  | VIC : TCAATGTGAATATGGGATAA |

a F : Forward primer, R : Reverse primer


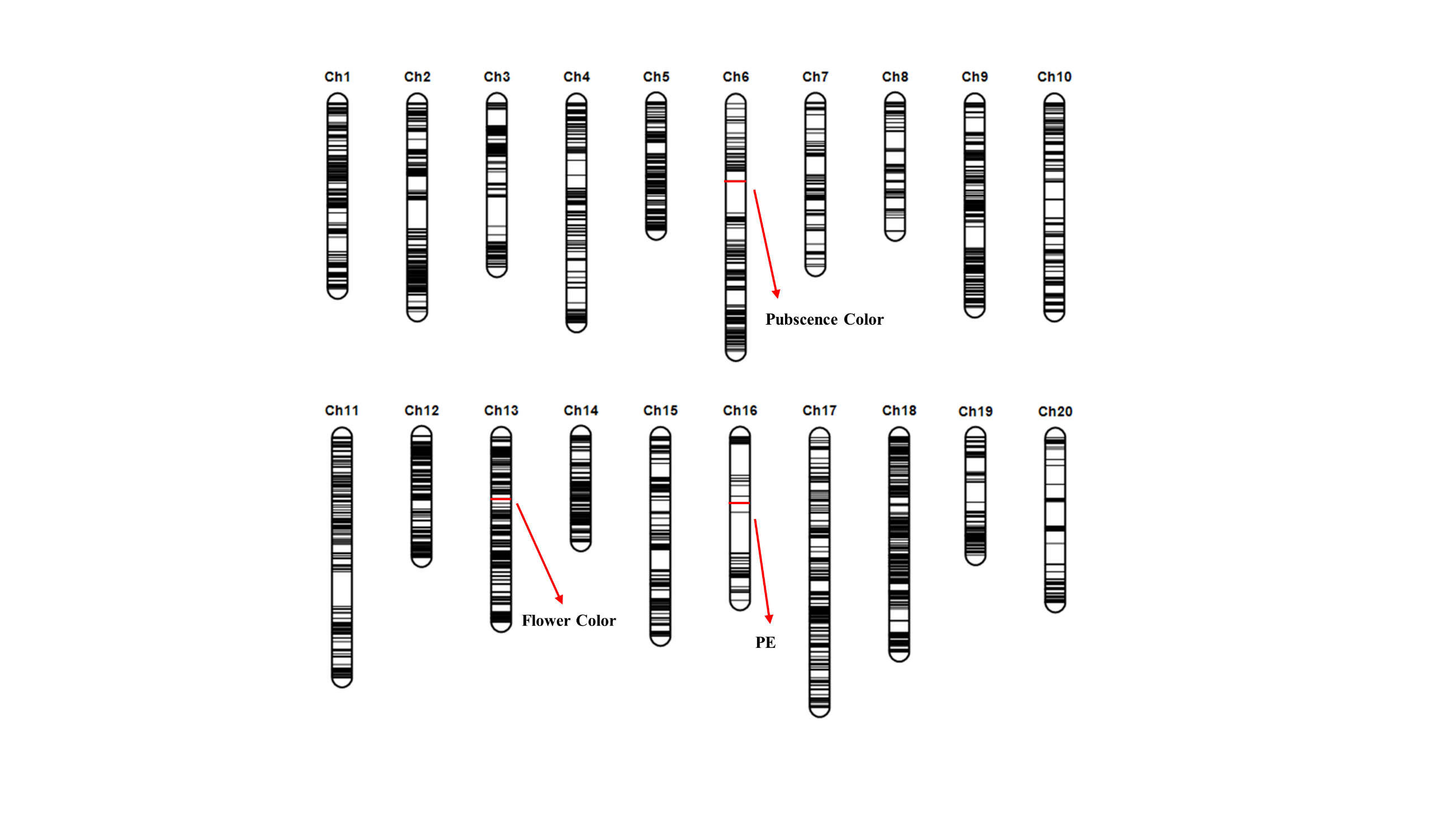


Figure S1. High saturated genetic map from RIL population cross from Daepung × Danbaek. Black bars mean SNP marker position on each chromosome. Red bars are locus associated with pubescence color (*T*) in Chromosome 6, flower color (*W1*) in chromosome 13 and phytotoxicity to etofenprox (PE) in chromosome 16.


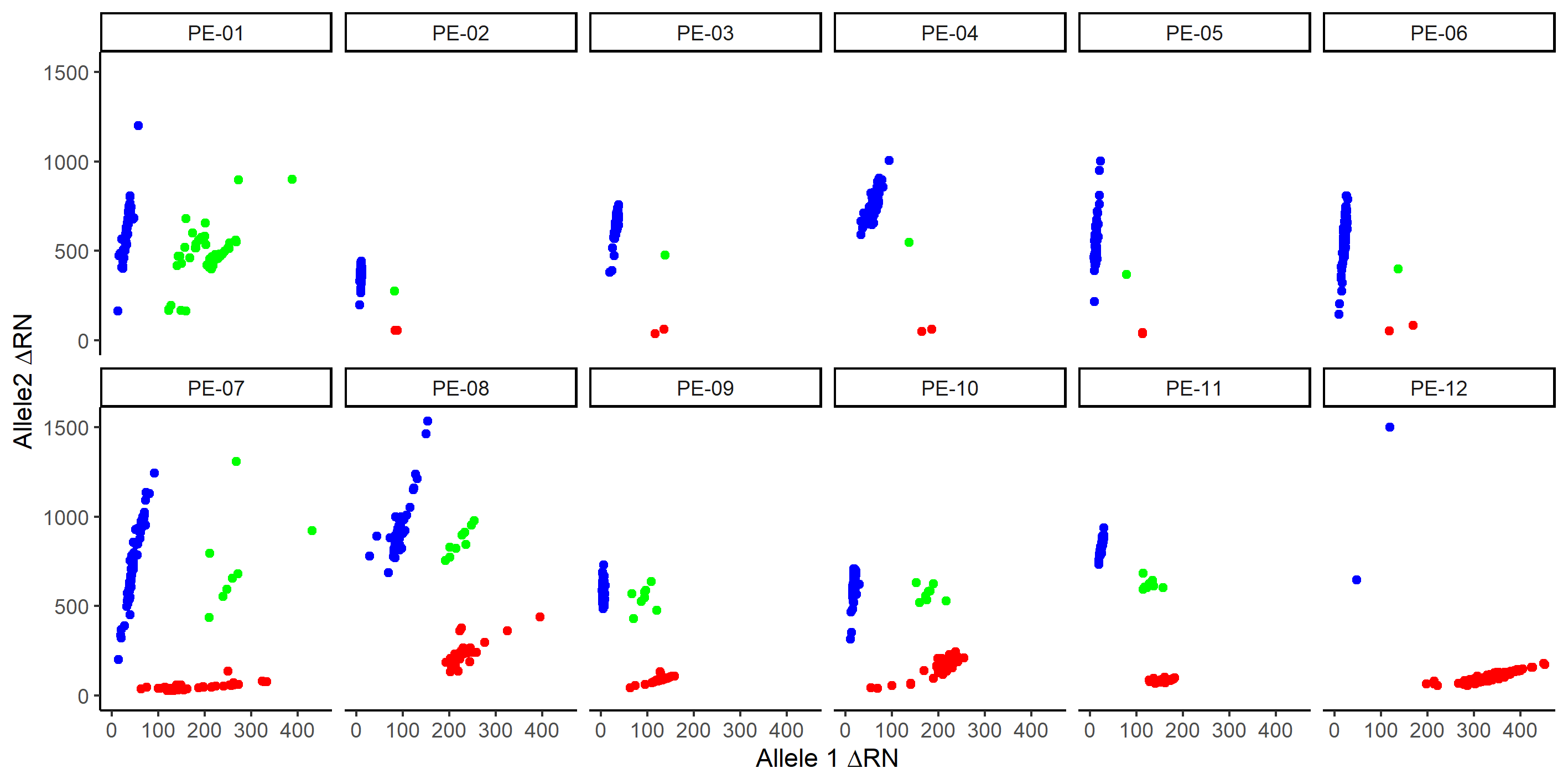


Figure S2. Genotyping results for TaqMan based SNP assay with the twelve PE markers to RIL population cross from 5002T × Kwangan. The scatter plots with x and y axes represent allele discrimination of the genotypes. The red and blue dots represent the homozygous alleles of 5002T and Kwangan, respectably and the green dots represent the heterozygous alleles.


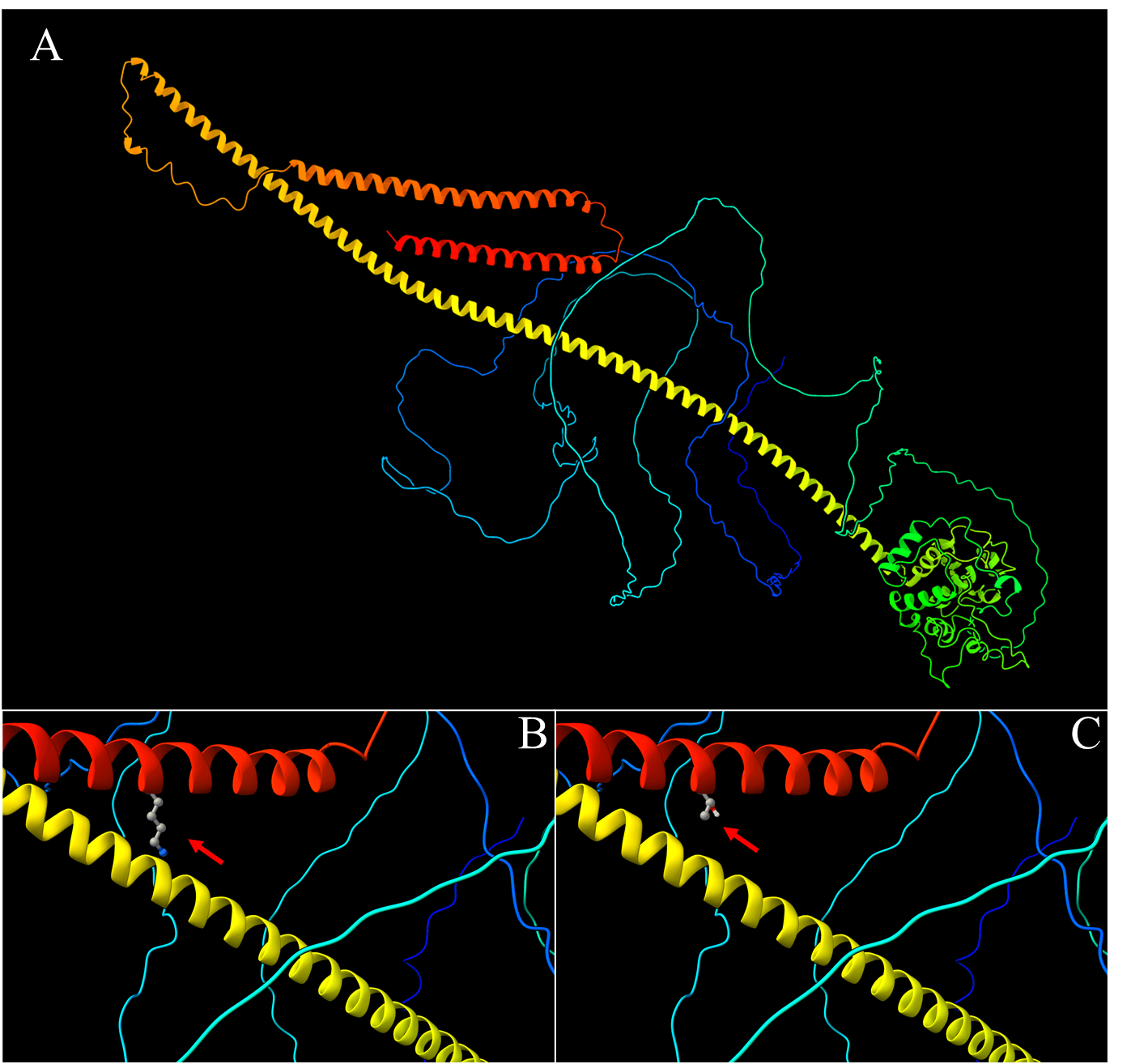


Figure S3. Predicted tertiary protein structure and energy minimized model of *Glyma.16G182200*. There are one nonsynonymous SNPs, which alter the amino acid sequence. This SNP was employed and *Glyma.16G182200* in Danbaek were predicted. Residue is pointed by red arrow (A) The tertiary protein model of *Glyma.16G182200 predicted* from Alphafold. (B) The enlarged image of *Glyma.16G182200* in Daepung without SNP mutation (C) The enlarged image of *Glyma.16G182200* in Danbaek with SNP mutation. The image is colored by blue to red from N-terminal to C-terminal.
